# Cause‐specific mortality in HPV+ and HPV− oropharyngeal cancer patients: insights from a population‐based cohort

**DOI:** 10.1002/cam4.1264

**Published:** 2017-11-24

**Authors:** Cecilie Nørregaard, Christian Grønhøj, David Jensen, Jeppe Friborg, Elo Andersen, Christian von Buchwald

**Affiliations:** ^1^ Department of Otorhinolaryngology, Head and Neck Surgery and Audiology Rigshospitalet University of Copenhagen Copenhagen Denmark; ^2^ Department of Oncology Rigshospitalet University of Copenhagen Copenhagen Denmark; ^3^ Department of Oncology Herlev Hospital University of Copenhagen Copenhagen Denmark

**Keywords:** Cause of death, human papillomavirus, oropharyngeal cancer

## Abstract

Identifying the causes of death in head and neck cancer patients can optimize follow‐up and therapeutic strategies, but studies in oropharyngeal squamous cell carcinoma (OPSCC) patients stratified by HPV status are lacking. We report cause‐specific mortality in a population‐based cohort of patients with OPSCC. Patients who had been diagnosed with OPSCC (*n* = 1541) between 2000 and 2014 in eastern Denmark were included in the study. Causes of death were collected through medical files and the Danish National Cause of Death registry. Deaths were grouped as (1) primary oropharyngeal cancer, (2) secondary malignancies, (3) cardiovascular and pulmonary disease, or (4) other/unspecified. The cumulative incidence of death and specific causes of death were determined using risk analysis. At follow‐up, 723 (47.5%) patients had died. The median time to and cause of death were determined: oropharyngeal cancer (*n* = 432; 1.00 year), secondary malignancies (*n* = 131; 2.37 years), cardiovascular and pulmonary causes (*n* = 58; 3.48 years), and unspecified causes (*n* = 102; 3.42 years). HPV/p16 status was the strongest predictor of improved survival across all causes of death. The only cause of death to decrease in incidence over the 2 years after treatment was death from OPSCC. HPV/p16 positivity was an independent factor for improved survival across all causes of death in patients with OPSCC. In addition, both HPV‐positive and HPV‐negative OPSCC patients faced high 5‐ and 10‐year mortality rates. Implementing secondary screening and prevention strategies for late toxicity and mortality are major goals in managing the treatment of these patients.

## Introduction

In eastern Denmark, as in the rest of the Western world, the incidence of oropharyngeal squamous cell carcinomas (OPSCC) is increasing, mainly due to the increase in a subset of tumors associated with human papillomavirus (HPV) infection 1, 2, 3, 4. This group of younger patients displays a better overall and progression‐free survival as well as survival after progression compared with the group of cancers induced by smoking and alcohol (e.g., HPV‐negative) 5. Smoking and alcohol use are causative factors for developing OPSCC, and it is likely that competing diseases might be another cause of death in this group, for example, cardiovascular or pulmonary diseases. Furthermore, smoking and alcohol intake may lead to the development of secondary malignancies in the head and neck region, upper digestive tract, and the respiratory system.

Although the improved survival of patients with HPV‐positive tumors is well demonstrated, little is known about the actual causes of death. A recent study comprising 4245 deaths in 5905 patients treated for OPSCC reported that the most common cause of death after the primary malignancy was cardiopulmonary diseases followed by secondary malignancies 6, 7, 8, 9, 10, but the timing and HPV status were not reported. In contrast, another study failed to show an increased risk of death due to cardiovascular disease or secondary malignancies in oropharyngeal cancer patients 7.

It remains unclear if and how HPV status and short‐term toxicity of OPSCC treatment impact mortality. Likewise, knowledge is lacking on the long‐term outcomes of HPV‐positive patients because these patients are more likely to become long‐term survivors and thus have a higher risk of experiencing late toxicity. Since HPV‐positive OPSCC patients typically are younger and have a lower consumption of alcohol and tobacco, they might have a lower risk of cardiopulmonic disease and secondary malignancies 11, 12.

Identifying the primary causes of death can help optimize follow‐up and therapeutic strategies for HPV‐positive and HPV‐negative OPSCC patients. We assessed causes of death in HPV‐positive and HPV‐negative oropharyngeal cancer patients from a population‐based setting in eastern Denmark.

## Methods

Patients diagnosed with OPSCC in eastern Denmark from 2000 to 2014 were included 3, 4, 13. Aside from the tonsillar and base‐of‐tongue cases, nonspecific tonsillar squamous cell carcinomas (e.g., soft palate) were included in the study. The cohort initially consisted of 1541 patients. When collecting data on the causes of death, the number was reduced to 1521 because of losses to follow‐up. The patients were identified through the Danish Head and Neck Cancer group (DAHANCA) database and validated through the national Danish Pathology Data Registry (DPDR) 3, 4. An expert head and neck pathologist confirmed the diagnosis of OPSCC from a hematoxylin and eosin (H&E)‐stained section of each tumor. The p16 staining was considered positive if a strong and diffuse nuclear and cytoplasmic reaction was present in more than 75% of the tumor cells 14. Formalin‐fixed paraffin‐embedded (FFPE) tumor specimens were handled according to standard operating procedures. Immunohistochemistry for p16 was carried out using the Ventana Benchmark Ultra autostainer with the UltraView detection kit and the p16 monoclonal antibody E6H4 with CC1 (Roche, Tucson, USA). DNA was isolated from two to four 10‐*μ*m sections using the DSP DNA Mini Kit and the QIAsymphony SP kit (Qiagen, Hilden Germany), according to the manufacturer's instructions. HPV‐DNA PCR was performed using the general primers GP5+/6+ and Platinum Taq DNA polymerase (Invitrogen, Naerum, Denmark). All samples with a negative GP5+/6+ PCR were subject to a GAPDH (housekeeping gene) PCR to confirm DNA quality 4, 13. Tumors were deemed truly HPV‐positive when they were HPV+/p16+.

Date of death and cause of death were collected from each patient's medical file and the Danish Register of Causes of Death (DRCD) 15. Data from DRCD were cross‐checked in the medical file when the information was implausible (e.g., the cause of death registered as an “accident,” even though the patient had been admitted to a hospital due solely to pneumonia). Data from the medical file were used as the most plausible information. If neither the medical file nor data from the DRCD were available, the cause of death was categorized as “unspecified.” Medical files were located for 540 patients and information from the DRCD was obtained from 581 patients; thus, 41 patients’ data were only available through the DRCD. In total, 157 patients’ files contained missing data. The DRCD is updated once a year and due to a validating process of the data, the registration is performed retrospectively 15. Patients were followed up until 1 October 2016. In Table 3 , 709 deaths are listed. The remaining 14 deaths were lost to follow‐up.

### Statistical analysis

Patients were categorized into four groups based on the cause of death: oropharyngeal cancer, cardiovascular or pulmonary causes, secondary malignancy, and unspecified causes. The cumulative incidences of death in the four groups were estimated by the Aalen‐Johansen method 16. The Cox regression model was used to calculate the effect of various covariates on risk of death in the four subgroups. The main model included the following covariates: curative treatment as two time‐dependent variables indicating either current or completed treatment (in contrast to no treatment or palliative treatment), age at diagnosis, year of diagnosis, gender, HPV‐DNA by PCR, p16 scoring, smoking (pack‐years), and UICC7 staging. Assumptions of linear effect of quantitative covariates and proportional hazards were applied. We analyzed the data using Statistical Analysis Software (SAS), version 9.4. This study was approved by the Danish DPA and Science Ethics Committee.

## Results

### Patient characteristics

A total of 1541 patients were diagnosed with OPSCC between 2000 and 2014 in eastern Denmark. After completing the data collection, the cohort consisted of 1521 patients, of whom 723 (47.5%) died during follow‐up (Tables 1 and 2). The median age was 59 years (HPV+:58.3 years; HPV−: 60.1 years) at diagnosis, and the median time of follow‐up for surviving patients was 4.88 years (0.25–16.73 years). Most patients were males (*n* = 1,117; 73.4%) and 826 (54.3%) were HPV/p16‐positive. Tobacco use was high with a median of 30 pack‐years. Based on the UICC7 classification scheme, 1205 (79.2%) patients had stage III or IV OPSCC. For the HPV/p16‐positive group, based on the UICC8 classification scheme, the majority had stage I and II OPSCC (*n* = 808; 53.1%). The majority of patients had a performance status of zero (*n* = 772; 50.8%) or one (*n* = 282; 18.5%). The most common treatment modality was radiotherapy (RT) (*n* = 783; 51.5%) followed by RT with concurrent chemotherapy (*n* = 613; 40.3%). Ninety‐one (6%) patients received palliative treatment or no treatment at all.

**Table 1:** Characteristics of patients treated for OPSCC in eastern Denmark between 2000 and 2014 (*n* = 1541)

	Median	Absolute numbers
*Age*	60 years	
Gender		
Female		424
Male		1117
Missing data		0
Status at follow‐up (dead/alive)		
Alive		801
Dead		738
Missing data		2
*Pack‐years*	30 pack‐years	
Pack‐years category		
0 pack‐years		341
0–10 pack‐years		111
11–20 pack‐years		151
More than 20 pack‐years		818
Missing data		120
HPV status		
HPV‐positive		826
HPV‐negative		675
Missing data		40
Stage UICC7		
Stage 0		0
Stage I		46
Stage II		168
Stage III		345
Stage IV A		860
Stage IV B		81
Stage IV C		32
Missing data		9
Treatment modality		
Radiotherapy		783
Radiotherapy + Chemotherapy		613
Palliative treatment		49
No treatment		42
Surgery		24
Other		11
Missing data		19
Performance score		
0		772
1		282
2		71
3		27
4		4
Missing data		385

**Table 2:** Characteristics of patients treated for OPSCC in eastern Denmark between 2000 and 2014 by HPV status (*n* = 1541)

	HPV‐positive	HPV‐negative	Absolute numbers
*Age (median)*	59	61	
Gender			
Female	203	214	417
Male	623	461	1084
Missing data			40
Status at follow‐up (dead/alive)			
Alive	639	162	801
Dead	286	454	740
Missing data			0
*Pack‐years (median)*	17	41	
Pack‐years category			
0 pack‐years	268	57	325
0–10 pack‐years	82	28	110
11–20 pack‐years	94	55	149
More than 20 pack‐years	325	474	799
Missing data			158
Stage UICC7			
Stage 0	0	0	0
Stage I	20	24	44
Stage II	73	91	164
Stage III	174	161	335
Stage IV A	494	346	840
Stage IV B	49	29	78
Stage IV C	10	20	30
Missing data			50
Treatment modality			
Radiotherapy	368	396	764
Radiotherapy + Chemotherapy	411	187	598
Palliative treatment	11	37	48
No treatment	10	31	41
Surgery	11	11	22
Other	10	0	10
Missing data			58
Performance score			
0	516	239	755
1	82	196	278
2	15	55	70
3	4	23	27
4	1	3	4
Missing data			407

### Causes of death in HPV‐positive and HPV‐negative patients

Of the total cohort of deceased patients, 222 (31.3%) were HPV/p16 positive (Table 3). In this group, the most common cause of death was primary OPSCC (*n* = 129; 58.1%), followed by secondary malignancies (*n* = 37; 16.7%) and unspecified causes (*n* = 32; 14.4%). Twenty‐four (10.8%) died from cardiovascular or pulmonary diseases. For the HPV/p16‐negative patients, the most common cause of death was primary OPSCC (*n* = 295; 60.6%), followed by secondary malignancies (*n* = 92; 18.9%) and unspecified causes (*n* = 67; 13.8%). Of the HPV‐negative patients, 34 (6.7%) died from cardiovascular or pulmonary diseases.

**Table 3:** Cause of death stratified by HPV status and mortality rates

Cause of death	No. of deaths			Time of occurrence (years)median (range)	5‐year mortality (95% CI)	10‐year mortality (95% CI)
	HPV‐positive	HPV‐negative	Total			
Oropharyngeal cancer	129	295	424	1.00 (0.02–14.02)	27 (0.24–0.3)	32 (0.3–0.35)
Secondary cancer	37	92	129	2.37 (0.076–14.82)	8 (0.07–0.1)	12 (0.1–0.14)
Cardiovascular and pulmonary diseases	24	33	57	3.47 (0.08–14.82)	3 (0.02–0.04)	5 (0.04–0.07)
Other/unspecified cause of death	32	67	99	3.24 (0.03–15.27)	5 (0.04–0.06)	9 (0.07–0.11)
Total	222	487	709			

### Timing of death and influence of patients and disease characteristics

**Figure 1:**
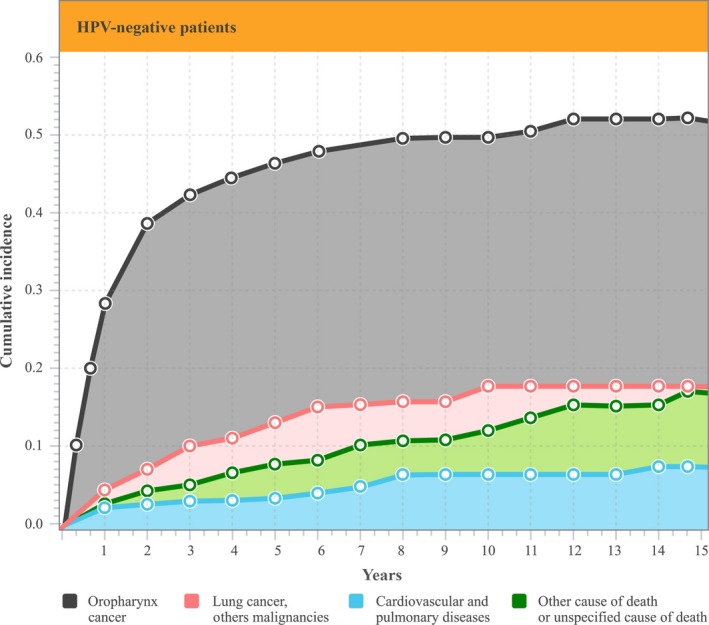
Cumulative incidence of death stratified by cause in HPV‐negative patients

**Figure 2:**
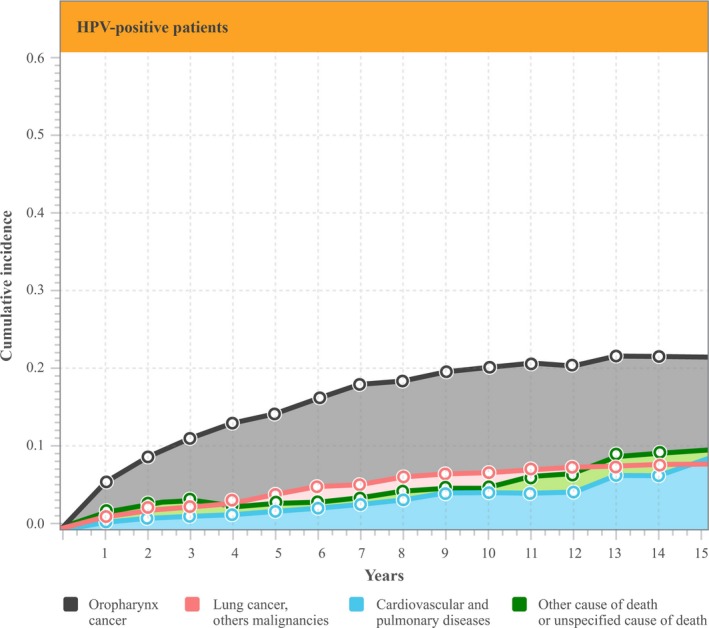
Cumulative incidence of death stratified by cause in HPV‐positive patients

In the total cohort of 723 patients who had died, 434 (59%) patients died within the first 2 years of OPSCC diagnosis, of whom 99 (22.8%) were HPV‐positive. Of the 434 patients, 315 (73%) (*n* = 72 HPV‐positive patients) died due to the primary oropharyngeal cancer, followed by 51 patients (12%) (*n* = 8 HPV‐positive patients) who died due to secondary malignancies. Death due to the primary oropharyngeal cancer decreased over time from 73% in the first 2 years to 23% in the remaining 5 years (from year 2 to 7) (*P < *0.01) (Figs. 1 and 2), most prominently in the HPV‐negative group. There were no significant differences in the remaining three subgroups when comparing the hazards ratio of dying within the first 2 years following diagnosis versus within 2–7 years. In addition, when stratified by cause of death, there was a 27% and 32% risk of dying from the primary OPSCC, respectively, with a 5‐ and 10‐year mortality rate of 8% and a 12% risk of dying from a secondary primary cancer (Table 3).

In the subgroup of patients who died from primary oropharyngeal cancer, the risk of death was influenced by several patient and disease‐specific characteristics (Table 4). In a multivariate analysis, the risk of death was higher among HPV−/p16− patients, males, smokers with a high number of pack‐years, older age, and those diagnosed with OPSCC at advanced stages (Table 4). Similar results were observed in the other three subgroups.

**Table 4:** Multivariate analysis on cause of death

Category	Index	OPSCC		CP		SM		UCD	
		HR	*P*	HR	*P*	HR	*P*	HR	*P*
Curative treatment		0.31	<0.0001	0.94	0.93	0.34	0.004	0.72	0.59
Age		1.02	<0.0001	1.09	<0.0001	1.04	<0.0001	1.04	0.0004
Year of diagnosis		0.97	0.01	0.97	0.53	0.88	<0.0001	1	0.1
Gender	Female	0.76	0.02	0.66	0.2	0.79	0.29	1.06	0.79
HPV/p16 status	HPV+/p16+	0.24	<0.0001	0.39	0.005	0.2	<0.0001	0.2	<0.0001
HPV/p16 status	HPV+/p16−	0.51	<0.0001	0.28	0.22	1.11	0.73	1.14	0.74
HPV/p16 status	HPV−/p16+	0.48	<0.0001	1.25	0.61	0.34	0.01	0.62	0.21
Pack‐years		1.01	<0.0001	1.01	0.19	1.01	0.09	1.002	0.61
UICC7	Stage I	0.07	<0.0001	0.68	0.71	0.11	0.01	0.47	0.22
UICC7	Stage II	0.15	<0.0001	1.46	0.63	0.46	0.06	0.46	0.13
UICC7	Stage III	0.27	<0.0001	0.95	0.94	0.38	0.01	0.43	0.08
UICC7	Stage IV	0.45	<0.0001	0.82	0.79	0.45	0.02	0.51	0.13

Category references: Gender, male; HPV/p16 status, HPV−/p16−; UICC7, Stage IV B and C. OPSCC, oropharyngeal squamous cell carcinoma; CP, cardiovascular and pulmonary diseases; SM, secondary malignancies; UCD, unspecified cause of death.

## Discussion

To our knowledge, this is the first study to report causes of death for HPV‐positive and HPV‐negative patients treated for OPSCC. Our results indicate that being HPV and p16 positive is a strong predictor of favorable prognosis across all causes of death. Most patients who died from primary OPSCC died within the first 2 years of diagnosis, and only 9% (*n* = 38) of the patients died after 5 years. We observed a 5‐ and 10‐year mortality rate of 27% and 32%, respectively, due to primary OPSCC. The HPV‐ and p16‐negative patients showed a greater risk of dying across all causes of death compared to the HPV‐positive patients.

Van Monsjou et al. reported that OPSCC patients have an increased risk of dying from secondary cancers outside the head and neck region 6. They also observed a significantly higher risk of dying from pneumonia, cardiovascular disease, gastrointestinal disease, or suicide. Baxi et al. confirmed that head and neck cancer patients have an increased risk of dying from other malignancies and cardiovascular diseases 7. However, neither of these studies reported data on HPV or p16 status. In the present study, information on the patients’ HPV/p16 status allowed precise stratification to analyze causes of death based on HPV status.

Screening for secondary malignancy is an important factor to reduce mortality, especially when considering the high level of tobacco use in the HPV‐negative population. In our study, we found a 10‐year mortality rate of 12% due to a secondary malignancy. Surprisingly, intensifying the frequency of screenings does not seem to affect the number of detected lung cancers, although prolonging the screening period may affect lung cancer detection rates 17. Our data indicate that late secondary malignancies, namely, lung cancers, are diagnosed frequently in patients with OPSCC. In a study from 2007 reporting causes of death in patients with oral and oropharyngeal cancer, the primary cause of death was recurrence of the primary cancer, followed by second primary cancers 18. Based on this finding, the authors recommend more frequent follow‐ups to increase detection of recurrence and secondary cancers. Our study shows a reduced risk of dying from secondary malignancies as well as cardiovascular and pulmonary diseases among HPV/p16‐positive patients compared to the HPV‐negative subgroup. This finding is important because it adds to the knowledge regarding the differences among HPV‐positive and HPV‐negative groups and supports the need to individualize follow‐ups based on the patient's HPV status.

The strength of our study is the population‐based design with long‐term follow‐up with only a small number of patients lost to follow‐up. Furthermore, we included important clinical and nonclinical parameters including HPV/p16 status. The treatment of OPSCC has changed little over time, making the data from this cohort uniform. We acknowledge certain limitations to the study. Since doctors who are not necessarily familiar with the patients’ medical background register the cause of death in the DRCD, the cause of death could be incorrectly registered. For instance, if the OPSCC patient suffered from secondary malignancies, this secondary malignancy may be registered as the primary cause of death, even though the actual cause is the OPSCC. Likewise, some patients were registered as dying of their primary OPSCC 14 years after the diagnosis, which is highly unlikely. Notably, this concerns a minority of the patient cohort.

In conclusion, patients with HPV‐positive OPSCC face a lower risk of dying from all causes of death when compared to the HPV‐negative OPSCC patients. Therefore, implementing secondary screening and prevention strategies for recurrence of the primary cancer and aiding in lifestyle changes to improve recovery are major treatment goals. The findings of our study indicate that being HPV and p16 positive is an independent factor for improved survival across all causes of death, although the 5‐ and 10‐year mortality rates for both HPV‐positive and HPV‐negative patients are high. Awareness of the patient's HPV status may aid in optimizing and individualizing follow‐ups in OPSCC patients.

## Conflict of Interest

None declared.

## Supporting information


**Table S1.** Detailed overview of the final cause of death stratified by HPV status.
