# Supplementary material for: Cause‐specific mortality in HPV+ and HPV− oropharyngeal cancer patients: insights from a population‐based cohort
Source: Cancer Med. 2017 Nov 24;7(1):87–94. doi: 10.1002/cam4.1264 (PMC5773974; doi:10.1002/cam4.1264)
Supplement: Supplementary file 1 — Table S1. Detailed overview of the final cause of death stratified by HPV status. [file CAM4-7-87-s001.docx]

**Supplementary table 1: Detailed overview of the final cause of death stratified by HPV-status**

|  | | HPV-status |  |
| --- | --- | --- | --- |
|  |  | HPV-positive | HPV-negative |
| Status at follow-up (dead/alive) | Alive | 598 | 178 |
|  | Dead | 227 | 496 |
| Final Cause of Death | Alive | 598 | 178 |
|  | Oropharyngeal cancer | 132 | 299 |
|  | Head and Neck malignancies other than OPSCC | 2 | 43 |
|  | Lung cancer | 16 | 29 |
|  | Gastrointestinal malignancies | 12 | 17 |
|  | Non-malignant gastrointestinal diseases | 3 | 7 |
|  | Alcoholic liver cirrhosis | 3 | 9 |
|  | Hematological diseases | 2 | 2 |
|  | Urinary tract malignancies | 3 | 2 |
|  | CNS diseases and malignancies | 8 | 8 |
|  | Breast cancer | 1 | 3 |
|  | Unknown primary tumor | 3 | 1 |
|  | Acute anemia caused by bleeding | 2 | 5 |
|  | Cardiovascular diseases | 13 | 16 |
|  | Chronic obstructive pulmonia | 5 | 6 |
|  | Lung infection | 6 | 11 |
|  | Cause of death unspecified | 14 | 36 |
|  | Suicide | 3 | 1 |
|  | Accident | 0 | 1 |
